# Cyp2c44 epoxygenase-derived epoxyeicosatrienoic acids in vascular smooth muscle cells elicit vasoconstriction of the murine ophthalmic artery

**DOI:** 10.1038/s41598-021-98236-w

**Published:** 2021-09-21

**Authors:** Jiong Hu, Marco Sisignano, Roman Brecht, Natarajan Perumal, Carlo Angioni, Iris-Sofia Bibli, Beate Fisslthaler, Hartmut Kleinert, Norbert Pfeiffer, Ingrid Fleming, Caroline Manicam

**Affiliations:** 1 Department of Ophthalmology, University Medical Centre of the Johannes Gutenberg University Mainz, Langenbeckstr. 1, 55131 Mainz, Germany; 2 Institute for Vascular Signalling, Centre for Molecular Medicine, Goethe University, Frankfurt, Germany; 3 German Centre of Cardiovascular Research (DZHK), Partner site RheinMain, Frankfurt, Germany; 4 Pharmazentrum Frankfurt/ZAFES, Institute of Clinical Pharmacology, University Hospital, Goethe-University, Frankfurt, Germany; 5 Fraunhofer Institute for Translational Medicine and Pharmacology ITMP, Frankfurt, Germany; 6 Department of Pharmacology, University Medical Centre of the Johannes Gutenberg University Mainz, Mainz, Germany

**Keywords:** Immunohistochemistry, Glaucoma, Animal disease models

## Abstract

Cytochrome P450 (CYP) signalling pathway has been shown to play a vital role in the vasoreactivity of wild type mouse ophthalmic artery. In this study, we determined the expression, vascular responses and potential mechanisms of the CYP-derived arachidonic acid metabolites. The expression of murine CYP (Cyp2c44) and soluble epoxide hydrolase (sEH) in the wild type ophthalmic artery was determined with immunofluorescence, which showed predominant expression of Cyp2c44 in the vascular smooth muscle cells (VSMC), while sEH was found mainly in the endothelium of the wild type ophthalmic artery. Artery of Cyp2c44^−/−^ and sEH^−/−^ mice were used as negative controls. Targeted mass spectrometry-based lipidomics analysis of endogenous epoxide and diols of the wild type artery detected only 14, 15-EET. Vasorelaxant responses of isolated vessels in response to selective pharmacological blockers and agonist were analysed ex vivo. Direct antagonism of epoxyeicosatrienoic acids (EETs) with a selective inhibitor caused partial vasodilation, suggesting that EETs may behave as vasoconstrictors. Exogenous administration of synthetic EET regioisomers significantly constricted the vessels in a concentration-dependent manner, with the strongest responses elicited by 11, 12- and 14, 15-EETs. Our results provide the first experimental evidence that Cyp2c44-derived EETs in the VSMC mediate vasoconstriction of the ophthalmic artery.

## Introduction

The role of nitric oxide (NO) in the regulation of ocular perfusion pressure, particularly in the pathogenesis of glaucoma, which is associated with an increased vascular resistance that affects the optic nerve head circulation, has been a subject of considerable interest^1,2^. In line of these investigations, our retrospective study has demonstrated for the first time that besides the predominant involvement of NO, the cytochrome P450 (CYP) signalling pathway also plays an integral role in mediating the vasoreactivity of one of the least-studied albeit crucial ocular blood vessels, the ophthalmic artery^3^. The membrane-bound, heme-containing CYP enzymes are frequently referred to as the third pathway of the arachidonic acid metabolism, which leads to the generation of highly potent vasoactive arachidonic acid epoxides or epoxyeicosatrienoic acids (EETs)^4,5^. Since their initial identification in the early 1980s, these epoxide metabolites generated by the CYP enzymes have garnered growing attention and their role in the regulation of blood flow in various tissues has emerged a subject of extensive scrutiny^6–11^. Alterations in EET bioavailability have been linked to the development of endothelial dysfunction and could potentially lead to the pathogenesis of various disease conditions^12–15^.

Epoxyeicosatrienoic acids are synthesized as four distinct regioisomers composed of 5,6-, 8,9-, 11,12- and 14,15-EET, which are reported to elicit vasodilation in an equipotent manner in some vascular beds such as the renal, coronary and pulmonary arteries of different species including human^10,16–21^, while they are also shown to behave in a regio-selective manner in others^22,23^. Intriguingly, depending on the cell type that generates them, EETs were also found to possess vasoconstrictive properties in the pulmonary and renovascular beds^22,24–29^. These findings have triggered a remarkable paradigm shift in the direction of investigations in recent years. Cellular and tissue levels of the EETs are tightly regulated by the CYP enzymes that generate them as well as by the soluble epoxide hydrolase (sEH) that converts the epoxides to their corresponding vicinal diols^13,14^. There have been significant advances in research endeavours and accumulation of an impressive body of literature about the pathophysiological roles of CYP and sEH enzymes in the coronary, renal, pulmonary and cerebral vascular beds^11,14,23,30–34^, as well as in the retina^35,36^. However, little is known about the potential impact of this pathway in the pathogenesis of glaucoma attributed to altered vasoreactivity of the retrobulbar vasculature. To date, the evidence implicating a role for the CYP-sEH pathway in regulating the tone of the wild type (WT) mouse ophthalmic artery has been obtained in our previous study using non-specific pharmacological inhibitors in vitro^3^. However, these findings are still largely limited and hence, cannot provide any information about the CYP isoform involved and its mechanisms owing to the non-isoform-specific nature of the CYP inhibitors employed, which cannot block the release of preformed EET pools incorporated into membrane phospholipids^11,37^. Moreover, several of the commonly used CYP inhibitors are known to interfere with the activation of potassium ion (K^+^) channels and also attenuate the generation of reactive oxygen species (ROS) and EETs without discrimination^38,39^.

Therefore, the largely unknown aspects of the Cyp-sEH signalling pathway in the ophthalmic artery provided the impetus to further explore the vasoreactivity of the regioisomeric EETs, as well as to determine the expression and localization of Cyp2c44 and sEH in the present study. For this purpose, apart from the use of more selective pharmacological tools, the state-of-the-art mass spectrometry-based epoxide and diol profiling, Western blot analysis and immunofluorescence staining using mice with targeted deletion of the *CYP* and *sEH* genes (Cyp2c44^−/−^ and sEH^−/−^) were instrumental to assess the role of this pathway in the regulation of ophthalmic arterial tone.

## Results

### Expression of sEH and Cyp2c44 in the murine ophthalmic artery

**Figure 1:**
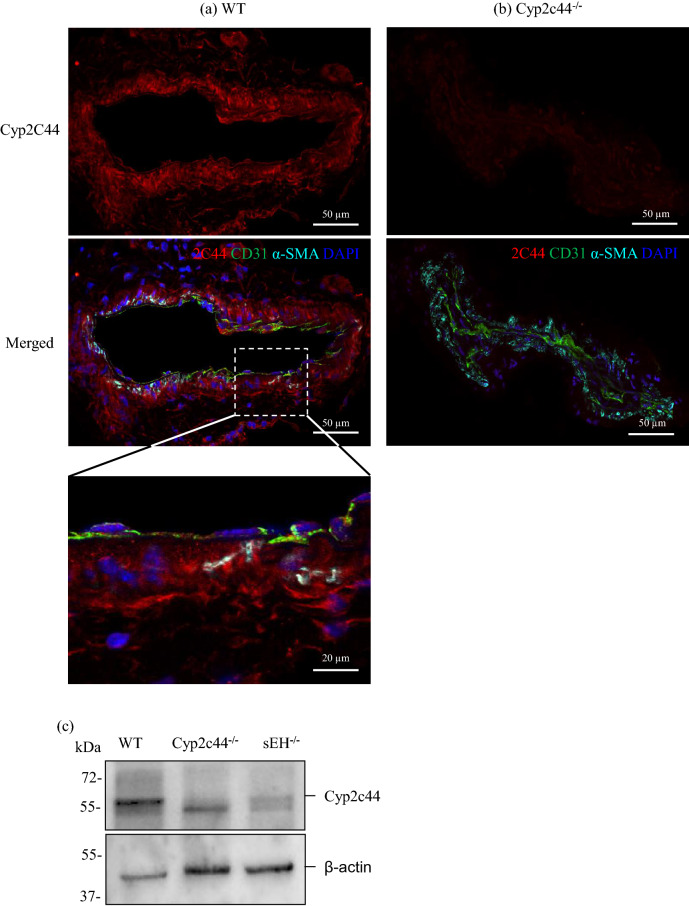
Expression and localization of Cyp2c44 in the mouse ophthalmic artery. Representative photomicrographs depicting the expression of Cyp2c44 in sagittal cryosections of the ophthalmic artery from (**a**) wild type (WT) and (**b**) Cyp2c44^−/−^mice using immunofluorescence staining. The magnified section of the artery shows the localization of this enzyme (in red) predominantly in the vascular smooth muscle cells stained with α-SMA (in cyan). Endothelial cells were stained green with CD31 and cell nuclei were stained blue with DAPI. (**c**) Western blot analysis of Cyp2c44 in the WT artery compared to Cyp2c44^−/−^ and sEH^−/−^ arterial samples. Cyp2c44 protein level was normalized to β-actin. Scale bars indicate 50 µm and 20 µm for lower and higher power magnification, respectively. Samples were pooled from n = 5 mice/ WT and n = 6 mice/ Cyp2c44^−/−^ and sEH^−/−^ each, per Western blot analysis. The Cyp2c44 and β-actin bands were cropped from full gels (Supplementary Fig. S1 online).

**Figure 2:**
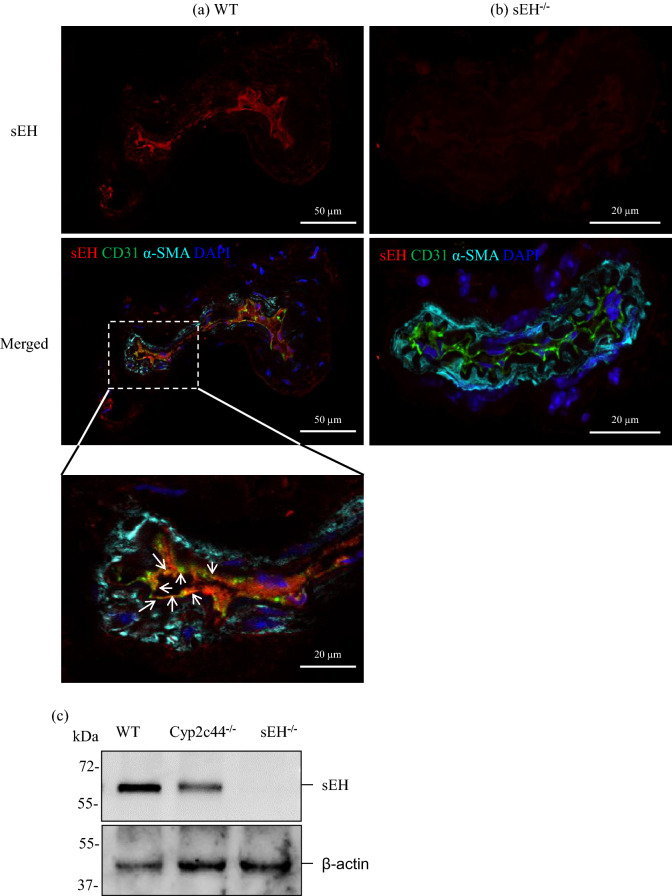
Expression and localization of sEH in the mouse ophthalmic artery. Immunofluorescence staining of representative ophthalmic artery sections demonstrated the expression of anti-sEH antibody (in red) in the (**a**) WT tissue and absence of its immunoreactivity in the (**b**) sEH^−/−^ artery, which served as a negative control. Endothelial cells were stained green with CD31, α-SMA in cyan and nuclei were stained blue with DAPI. The magnified section of the artery shows the localization of sEH in the endothelial cells, as indicated by arrows. (**c**) Representative blot of sEH expression in the artery samples demonstrated the expression of this protein in the WT artery compared to null expression in the sEH^−/−^ artery. The sEH protein level was normalized to β-actin. Scale bars indicate 50 µm and 20 µm for lower and higher power magnification, respectively. Samples were pooled from n = 5 mice/ WT and n = 6 mice/ Cyp2c44^−/−^ and sEH^−/−^ each, per Western blot analysis. The sEH and β-actin bands were cropped from full gels (Supplementary Fig. S1 online).

We sought to determine the presence and localization of sEH and Cyp2c44 in the cross-sections of the ophthalmic artery employing specific antibodies. Both enzymes were detected to be expressed in the WT mouse ophthalmic artery. The Cyp2c44 enzyme was predominantly detected throughout the entire vascular wall, including the vascular smooth muscle cells (Fig. 1a). The specificity of the signal was confirmed by the lack of fluorescence in arteries from Cyp2c44^−/−^ mice (Fig. 1b). Western blot analysis further confirmed the expression of this protein in the WT artery compared to null expression in the Cyp2c44^−/−^ samples (Fig. 1c). On the other hand, the expression of sEH was largely confined to the CD31-positive endothelium (Fig. 2a) compared to the absence of fluorescent signal in the artery of mouse with targeted deletion of the *sEH* gene, which was used as a negative control (Fig. 2b). Subsequent Western blot analysis also confirmed the expression of this enzyme in WT samples, whereas sEH gene disruption resulted in complete loss of expression (Fig. 2c). In all Western blot analyses, the artery samples were not analysed individually due to the very small amount of samples available per mouse, which has been a challenge for optimum protein extraction from this ocular vascular bed^40,41^. Hence, the samples were pooled from 5–6 mice per group to yield sufficient amount of proteins for analyses and the blots are representation of the expression of both enzymes. The Cyp2c44, sEH and β-actin bands were cropped from full gels (Supplementary Fig. S1 online).

### Profiles of eicosanoids and diols

To further assess the bioavailability of EETs and DHETs in the ophthalmic artery samples, a robust orthogonal analytical method using LC–MS/MS-based lipidomics approach was employed. Among the panel of the targeted individual lipid metabolites, only 14,15-EET could be quantified (0.60 ± 0.11 pg/ mg tissue), as tabulated in Table 1. All other eicosanoids were virtually undetectable and below the limit of quantification. The chromatograms of 14,15-EET are represented as three biological replicates of samples relative to the deuterated internal standard (Supplementary Fig. S2 online).

**Table 1:** Targeted quantification of Cyp epoxygenase- and sEH-derived arachidonic acid lipid metabolites in the ophthalmic artery.

Lipid mediator	Concentration (pg/mg)
5,6-EET	< LLOQ*
8,9-EET	< LLOQ*
11,12-EET	< LLOQ*
14,15-EET	0.60 ± 0.11
5,6-DHET	< LLOQ*
8,9-DHET	< LLOQ*
11,12-DHET	< LLOQ*
14,15-DHET	< LLOQ*

*LLOQ lower limit of quantification.

### Effects of arachidonic acid metabolites on the vasoreactivity of ophthalmic artery to acetylcholine

**Figure 3:**
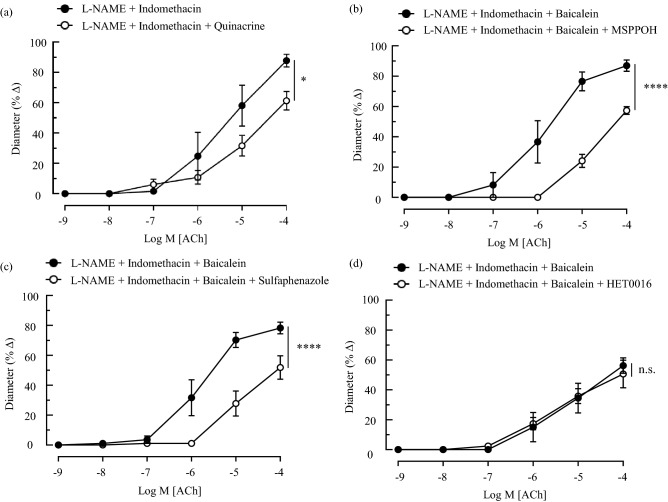
Cholinergic vasodilator responses of the ophthalmic artery to inhibition of the production of CYP-derived arachidonic acid lipid mediators. (**a**) Non-selective phospholipase inhibitor, quinacrine partially attenuated the vasodilator responses to ACh. The inhibition of CYP epoxygenase with 10 µmol/L of (**b**) MSPPOH and (**c**) sulfaphenazole resulted in marked blunting of the dilatory responses. However, the inhibition of CYP ω-hydroxylase with (**d**) HET0016 (1 µmol/L) conferred negligible inhibitory effects on ACh-induced vasodilation. Values are expressed as mean ± standard error of the mean (s.e.m), n = 6 per group. Absence of error bar indicates that the s.e.m was less than the size of the symbol. *P < 0.05 *vs.* L-NAME + indomethacin; ****P < 0.0001, n.s. non-significant, *vs.* L-NAME + indomethacin + baicalein.

To determine the effect of arachidonic acid released from the membrane phospholipids, ophthalmic arterial segments were incubated with the nonselective phospholipase inhibitor, quinacrine, in combination with L-NAME (100 µmol/L) and indomethacin (10 µmol/L). Subsequent cumulative administration of ACh (1 nmol/L–100 µmol/L) to preconstricted artery resulted in significant attenuation of vasodilation (L-NAME + indomethacin: 87.81 ± 4.21% *vs.* quinacrine: 61.30 ± 6.14%, *P* < 0.05) (Fig. 3a). Next, the inhibition of epoxygenases with MS-PPOH [L-NAME + indomethacin + baicalein (this combination blocking will be referred to as ‘reference’ henceforth): 86.89 ± 3.72% *vs.* MS-PPOH: 57.32 ± 2.59%, *P* < 0.01; Fig. 3b] and, specific inhibition of Cyp2c epoxygenases with sulfaphenazole (reference: 78.25 ± 3.90% *vs.* sulfaphenazole: 51.82 ± 7.83%, *P* < 0.01; Fig. 3c) demonstrated significant attenuation of the vasodilatory responses to ACh. Conversely, the inhibition of ω-hydroxylase with HET0016 caused negligible dilation (reference: 56.18 ± 5.16% *vs.* HET0016: 50.56 ± 9.22%; Fig. 3d), suggesting that the arachidonic acid metabolites generated via the Cyp ω-hydroxylase pathway, namely 19- and 20-HETEs, do not play a significant role in mediating the cholinergic vasodilator responses in this vascular bed.

**Figure 4:**
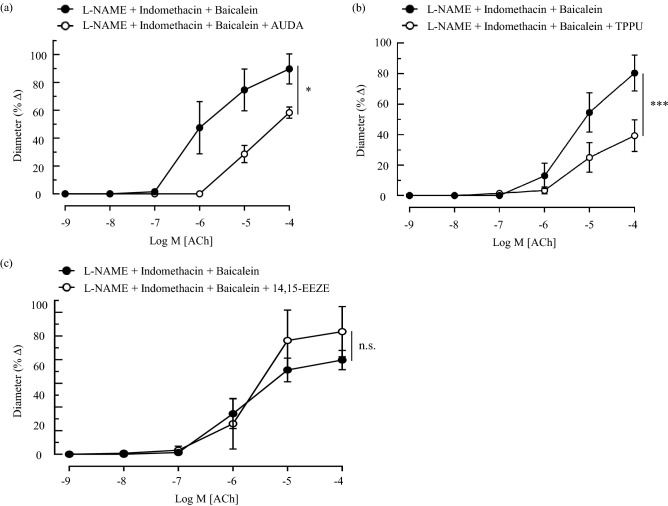
Changes in the vasoreactivity of ophthalmic artery in response to the inhibition of EET catalysis and direct antagonism of EETs. The administration of 100 nmol/L of sEH inhibitors comprising (**a**) AUDA and (**b**) TPPU resulted in significant reduction of vasodilation, whereas the selective inhibition of EETs with (**c**) 14, 15-EEZE (10 µmol/L) showed a noticeable increment in the pre-constricted vascular diameter albeit not statistically significant. Values are expressed as mean ± s.e.m, n = 6 per group. Absence of error bar indicates that the s.e.m was less than the size of the symbol. *P < 0.05; ***P < 0.001, n.s. non-significant, *vs.* L-NAME + indomethacin + baicalein.

The vasodilation elicited by ACh administration was significantly inhibited by both sEH inhibitors comprising AUDA (reference: 89.65 ± 10.77% *vs.* AUDA: 58.29 ± 4.03%, *P* < 0.05; Fig. 4a) and TPPU (reference: 80.37 ± 11.78% *vs.* TPPU: 39.33 ± 10.46%, *P* < 0.001; Fig. 4b). Subsequently, although the exposure of the ophthalmic artery to 14, 15-EEZE exhibited noticeable vasodilation in comparison to the control vessels, it did not reach statistical significance (reference: 79.65 ± 8.14% *vs.* 14, 15-EEZE: 103.65 ± 21.26%; Fig. 4c). It is, however, important to highlight that the observed vasodilatory response following the inhibition of the EETs with 14, 15-EEZE may be hinting to a possible inherent vasoconstrictive property of these lipid mediators.

### EET-induced contraction of the murine ophthalmic artery

**Figure 5:**
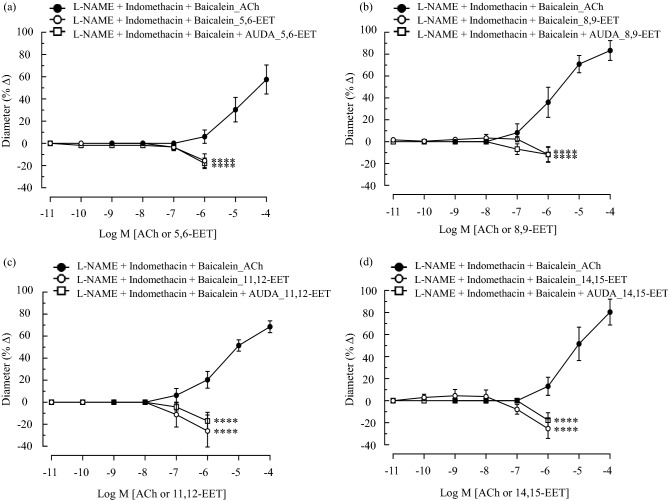
Exogenous administration of synthetic EET regioisomers evoked vasoconstriction of the ophthalmic artery. Cumulative application (0.01 nmol/L–1 µmol/L) of (**a**) 5, 6- (**b**) 8, 9- (**c**) 11, 12- and (**d**) 14, 15-EET in the absence and presence of AUDA (100 nmol/L) exhibited significant concentration-dependent vasoconstriction of the vessels. Values are expressed as mean ± s.e.m, n = 6 per group. Absence of error bar indicates that the s.e.m was less than the size of the symbol. ****P < 0.0001, *vs.* L-NAME + indomethacin + baicalein_ACh at respective concentration.

Based on our findings with specific inhibitors, the next set of experiments were carried out to test the hypothesis that EETs behave as vasoconstrictors. The effects of cumulative application of the EETs (0.01 nmol/L–1 µmol/L) on murine ophthalmic arterial rings sub-maximally contracted with phenylephrine were examined. Concentrations of EETs higher than 1 µmol/L were not used owing to the high cost and non-physiological concentrations of these lipids^26,29^. Remarkably, exogenous exposure to all regioisomers of EETs in the absence and presence of AUDA resulted in significant (*P* < 0.0001) concentration-dependent vasoconstriction of the vessels. All four regioisomers exhibited the same pattern of vasoconstriction and there was no significant difference in the vessels incubated with and without AUDA: 5, 6-EET (ACh: 6.06 ± 6.06% *vs.* EET: − 15.61 ± 6.22% *vs.* AUDA_EET: − 17.95 ± 4.75%, Fig. 5a), 8,9-EET (ACh: 35.98 ± 13.79% *vs.* EET: − 11.75 ± 7.18% *vs.* AUDA_EET: − 11.78 ± 6.61%; Fig. 5b), 11,12-EET (ACh: 20.35 ± 7.60% *vs.* EET: − 26.14 ± 14.62% *vs.* AUDA_EET: − 17.05 ± 7.90%; Fig. 5c), and 14,15-EET (ACh: 13.01 ± 8.28% *vs.* EET: − 25.40 ± 8.87% *vs.* AUDA_EET: − 17.72 ± 6.70%; Fig. 5d).

## Discussion

The current study characterized the signalling mechanisms that govern the CYP–mediated arachidonic acid metabolic pathway, with particular interest in the vasoreactivity of eicosanoids, EETs. The key findings emerging from our investigation demonstrated that Cyp2c44 is localized predominantly in the vascular smooth muscle cells and EETs behave as vasoconstrictors in the ophthalmic artery of WT mouse. Our study is among the very few indicating that EETs participate in vasoconstriction and the results are concordant with earlier studies in the rabbit renovasculature, and in the murine and rabbit pulmonary circulation^22,24–27,29^. In our current investigation, the exogenous administration of all regioisomers of EETs exhibited a similar pattern of contraction, with the strongest responses seen in arteries treated with 11, 12- and 14, 15-EETs. This finding comes as no surprise as these isomers have been repeatedly shown to elicit the greatest increase in perfusion pressure in other vascular beds^24–27,29^. Interestingly, targeted lipidomics analysis of endogenous levels of these lipid mediators in the ophthalmic artery could only quantify 14, 15-EET, while the other isomers were below detection level.

Epoxyeicosatrienoates are synthesized by different CYPs, mainly of the 2C and 2J subfamilies^42,43^. The expression levels, localization and potency of these isozymes differ in various tissues, and the propensity for generating certain EET subtypes are also species-dependent^30,44,45^. The predominant murine CYP is Cyp2c44^43,46^ and it has been identified to play key roles in mouse tissues such as retina, lung, hepatocytes, renal proximal tubules, cardiac tissue^36,46–49^, as well as in the ophthalmic artery in the present study. Immunofluorescent staining has clearly depicted the presence of Cyp2c44 in the VSMC of the ophthalmic artery and its expression has been confirmed employing Western blot analysis. The latter detected the presence of unspecific bands, which are attributed to the fact that the murine Cyp2c subfamily has the largest number of members compared to only four *CYP2C* genes in humans and nine in rats^50,51^. Similarly, rabbit lung microsomal proteins probed with anti-CYP2J antibody also showed two distinct bands, thereby suggesting that the CYP2J isoform may actually be multitudinous^29^ Nevertheless, we were able to clearly demonstrate that the upper band represented the Cyp2c44 protein in the present investigation with the use of artery samples from mice with targeted deletion of the Cyp2c44 gene that served as a negative control. Moreover, this is also the subtype with the lowest homology with other known murine Cyp2c enzymes, which raises an intriguing possibility that Cyp2c44 may possess unique physiological properties^14,48,52^. Hence, based on our findings, it is tempting to suggest that the expression pattern of Cyp2c44 in the ophthalmic artery mediates the synthesis of mainly the terminal epoxide 14,15-EET, while the other EET regioisomers could be by-products, which are synthesized in low amounts and cannot be detected with our approach^53^. On the other hand, it is well-recognized that EETs are rapidly hydrated by sEH following synthesis, leaving only the most abundant EET in levels that could be detected by MS^54–56^.

The apparent promiscuity of EETs is well-documented and as such, although only one isoform could be detected in our investigation, the contribution of other epoxyeicosanoids in vivo cannot be overlooked due to the synergistic, additive, or even counteracting effects among EET regioisomers^25^. Originally, most studies including ours, which assessed the potential involvement of EETs as secondary messenger molecules that mediate vasoreactivity, were based on the use of a plethora of CYP inhibitors such as 17-ODYA and miconazole to impede the production of endogenous EETs and/ or exogenous administration of sub-physiological concentrations of synthetic EETs owing to the labile nature of the CYP enzymes^3,14,57–59^. However, the effects of direct application of EETs may not be comparable to those caused by endogenous products of CYP due to the activation of a myriad non-specific downstream reactions by the use of high concentrations of synthetic EETs required to elicit the indicated biological responses^39,57^. A most germane finding to exemplify this observation is found in a study by Fleming et al., who have elegantly highlighted that the exogenous addition of EETs to endothelial cells mitigated the activation of NF-κB but the augmentation of endogenous CYP activity had resulted in contradictory outcomes^60^. Several studies also lend credence the provocative notion that most lipid mediators do not function as an isolated entity but are part of complex signalling pathways, which temporally and spatially control the biosynthesis of the putative autacoids to coordinate the observed cellular responses^25,57,58^. Therefore, the administration of synthetic lipid moieties to organ bath or cell culture is highly likely to bypass the integrated cellular response and interaction at multiple molecular levels that regulates cellular function in vivo. Albeit the accumulation of convincing experimental evidence from attempts made to better understand the functional significance of these observations attributed to EETs, more works remain to be done before we can elucidate these discordant findings without bias.

The complex crosstalk between these arachidonic acid pathways, which contributes to the variability in cell signalling and vasoactivity of the regioisomeric EETs has provided the impetus for developing highly specific blockers In our present investigation, we employed two inhibitors of CYP and sEH with divergent structural and mechanistic properties to rule out the assumption that most blockers inhibit the enzymatic reactions of all isoforms in a similar manner and ultimately, to provide unbiased evidence to support the observed vascular reactions. However, the use of epoxygenase inhibitors only addresses those EETs that would be produced upon stimulation by agonists such as ACh^61^. Remarkably, EETs do not necessarily have to be synthesized de novo on demand because a latent pool of pre-formed EETs is immediately available and directly liberated via the activation of phospholipase A_2_, which is independent of the CYP epoxygenase activity^30,62^. This phenomenon highlights the unique feature of the eicosanoids, which is their tendency to be avidly taken up by cells upon synthesis, esterified and re-incorporated into membrane phospholipid stores by an acyl coenzyme synthase^5,37^. The incorporation of these esterified lipids into phospholipid bilayers constitute a powerful mechanism of physiological importance to alter the properties of membrane lipid composition and fluidity, and initiate receptor-independent cell signalling pathways^14,54,63^.

**Figure 6:**
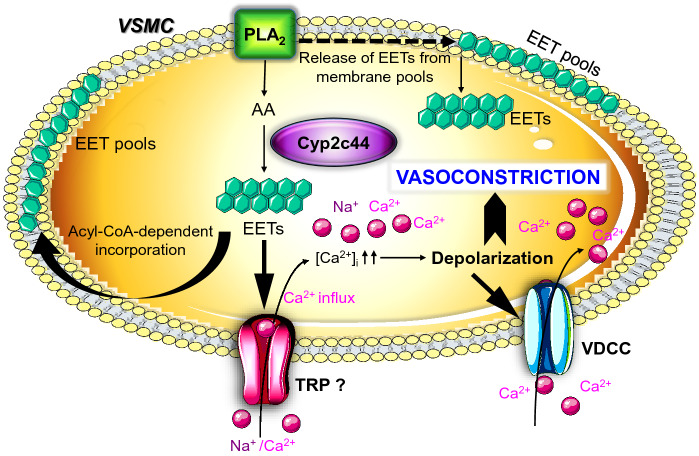
Schematic overview of the hypothesized mechanisms underlying CYP-mediated vasoconstriction on the murine ophthalmic artery. CYP localised in the vascular smooth muscle cell (VSMC) mediates the synthesis of EETs, which stimulate the activation and translocation of transient receptor potential (TRP) channel, resulting in elevated intracellular Ca^2+^ concentration [Ca^2+^]_i_. This then leads to membrane depolarization and activation of voltage-gated calcium channels and vasoconstriction. PLA_2_, Phospholipase A2; AA, arachidonic acid; TRP, transient receptor potential channel; VDCC, voltage-dependent calcium channel(s); Question mark represents the unknown TRP channel.

In our investigation, the inability of two mechanistically different CYP inhibitors to completely block the production of endogenous EETs and thereby, augment the vasodilator responses to ACh may be attributed to the sustained release and prevalent availability of EETs from membrane phospholipid pools. Hence, epoxygenase would not be required until the EET pools had been fully depleted^61,64^. It takes considerably longer to completely deplete the intracellular pool of EETs in tissues and eliminate myogenic tone after the administration of CYP inhibitors^65^. Moreover, the attenuation of the vasodilator response to ACh following CYP inhibition is an indication that any relaxant response may also have been dependent on epoxy derivatives other than those generated from arachidonic acid such as linoleic acid, docosahexaenoic acid and eicosapentaenoic acid^14^.Therefore, to ascertain that the illustrated results reflect the activity of EETs, we examined the effects of a selective and potent EET antagonist, 14, 15-EEZE, which does not interfere with the generation of CYP-derived ROS as well as does not discriminate between EETs synthesized on demand or released from a stored pool^7,26,58,61^. The findings emerging from this experiment demonstrated that the antagonism of the vascular effects observed in the ophthalmic artery occurs partially via the blockade of EET activity. Additionally, the cellular localization and expression of CYP are important determinants of the biological actions of EETs^66,67^. It has been reported that EETs stimulate the activation and translocation of transient receptor potential (TRP) channels in vascular smooth muscle cells leading to elevated intracellular Ca^2+^ concentration [Ca^2+^]_i_ and vasoconstriction, such as in the pulmonary vasculature^26,68^. A summary of a tenable hypothesis for the proposed mechanism by which EETs operate in the mouse ophthalmic artery based on the findings of the present study is presented Fig. 6.

Although our study has endeavoured to provide experimental evidence to elucidate the role of EETs as vasoconstrictors in this retrobulbar blood vessel, there are several limiting aspects. First, we were unable to completely measure the endogenous levels of all EET regioisomers using the MS-based lipidomics analysis due to the infinitesimal size of the ocular artery and hence, limited amount required to reach the measurable threshold; the drawbacks of which have also been the reminiscent of our previous MS-based proteomics studies^41,69^. Second, we are yet to selectively investigate the mechanisms of EETs-mediated vasoconstriction in this artery. . Nevertheless, the determination of the effects of incorporation of EETs into membrane lipids and their potential CYP epoxygenase-independent mechanism of vasoconstriction remains an endeavour for future studies to address the importance of vascular cellular EETs . Finally, we have not investigated the participation of other CYP subfamily enzymes and the exact mechanism(s) underlying vascular smooth muscle contraction owing to EETs generated by these CYPs remains to be elucidated and is a focus for future studies.

These drawbacks have certainly opened up potential caveats for future studies to test the hypothesis that EETs must be incorporated into membrane phospholipids to potentiate vasoconstriction, and also to identify the mechanisms underlying potential activation of the TRP channel(s) by EETs to elicit vascular smooth muscle contraction. Next, notwithstanding the use of samples from sEH^−/−^ and Cyp2c44^−/−^ mice, which has provided well-defined results for immunostaining and Western blot analysis in the present study, the employment of these mice will be valuable for further determination of the physiological role(s) of EETs or the lack thereof in mediating vasoreactivity in this vascular bed in the future.

Based on the findings outlined here, we can conclude that Cyp2c44-derived EETs localized predominantly in the vascular smooth muscle cells mediate vasoconstriction of the wild type murine ophthalmic artery in a concentration-dependent manner, with the highest level of endogenous regioisomer identified as the 14, 15-EET. Although the results of this study have far-reaching pathophysiological significance in glaucoma, EETs have an important role in maintaining the vascular tone of the ophthalmic artery that may consequently influence the perfusion pressure of the eye. To date, this remains the foremost interpretation of the arachidonic acid-derived epoxyeicosanoid signalling in this least-studies yet pivotal ocular arterial bed, which continues to be a subject for further investigations.

## Materials and methods

### Animals

All animal experimental procedures using mice were conducted in strict adherence to the Association for Research in Vision and Ophthalmology (ARVO) Statement for the Use of Animals in Ophthalmic and Vision Research and followed the recommendations in the Animal Research: Reporting of In Vivo Experiments (ARRIVE) guidelines (https://arriveguidelines.org). Animal care conformed to the institutional guidelines and the EU Directive 2010/63/EU for animal experiments. This study was approved by the institutional animal care committee [Translational Animal Research Centre (TARC)] of the University Medical Centre of the Johannes-Gutenberg University Mainz and animal use was in accordance with the 3R principle to replace, reduce and refine. Male C57BL/6 J mice (The Jackson Laboratory, Bar Harbour, ME, USA) aged 3–5 months old were used in this study. Both knockout mouse strains (sEH^−/−^ and Cyp2c44^−/−^) used for immunofluorescence staining and Western blot analysis were generated as described^36,70^. Originally, the sEH^−/−^ mice were provided by Dr. Frank Gonzalez (National Institutes of Health, Bethesda, MD, USA) and cross-bred for ~ 20 generations onto the C57BL/6 background in the animal house facility at Frankfurt University^70^. Floxed Cyp2c44 mice were generated by TaconicArtemis (Cologne, Germany) using C57BL/6 embryonic stem cells for gene targeting^36^. Floxed Cyp2c44 mice were crossed with Gt(ROSA)26Sortm16(Cre)Arte mice expressing Cre under the control of the endogenous Gt(ROSA)26Sor promoter (TaconicArtemis) to generate mice globally lacking Cyp2c44 (Cyp2c44^−/−^). Animals were housed under standardised conditions with12h light/ dark cycle, at temperature 23 ± 2 °C and humidity 55 ± 10%. Mice were provided standard mouse chow and water ad libitum.

### Chemicals and reagents

The following chemicals were used in Krebs–Henseleit buffer preparation (in mmol/L): NaCl, 118.3; NaHCO_3_, 25; glucose, 11; KCl, 4.7; CaCl_2_, 2.5; MgSO_4_, 1.2 and KH_2_PO_4_, 1.2 (all from Carl Roth GmbH, Karlsruhe, Germany). Drugs used in the vasoreactivity study are as follows: N^ω^-nitro L-arginine methyl ester (L-NAME), indomethacin, acetylcholine hydrochloride (ACh), phenylephrine and baicalein (Sigma-Aldrich Chemie GmbH, Steinheim, Germany), quinacrine, sulphaphenazole, N-(methylsulfonyl)-2-(2-propynyloxy) benzenehexanamide (MS-PPOH), N-(4-butyl-2-methylphenyl)-N'-hydroxy-methanimidamide (HET0016), 12-[[(tricyclo[3.3.1.13,7]dec-1-ylamino)carbonyl]amino]-dodecanoic acid (AUDA), N-[1-(1-oxopropyl)-4-piperidinyl]-N’-[4-(trifluoromethoxy)phenyl)-urea (TPPU), 14,15-epoxyeicosa-5(Z)-enoic acid (14,15-EEZE), and all four EET regioisomers i.e. ( ±)5,6-, ( ±)8,9-, ( ±)11,12-, and ( ±)14,15-EET were purchased from Cayman Chemical (Ann Arbor, MI, USA). Ethanol in 14, 15-EEZE and EETs was evaporated and dissolved in dimethyl sulfoxide (DMSO). L-NAME was dissolved in phosphate buffer saline (PBS), whereas indomethacin, baicalein, quinacrine, sulphaphenazole, MS-PPOH, TPPU and AUDA were dissolved in DMSO, according to the manufacturer’s instructions. DMSO at ≤ 0.2% (v/v) did not influence vasoreactivity, as reported previously^3,71,72^.

### Immunofluorescence

Ophthalmic artery were embedded in OCT compound (Tissue Tek/Sakura, Staufen, Germany), frozen at − 80 °C and cut into 10 µm thick sections. After fixation with 4% PFA for 15 min, sections were permeabilized with 1% BSA and 0.5% Triton X-100 in 1 × PBS for 1 h at room temperature. Then, sections were incubated with primary antibody and incubated overnight at 4 °C. The following antibodies were used: sEH (1:200, provided by Prof. Michael Arand, Zurich, Switzerland)^73^, Cyp2c44 (1:200, provided by Prof. Darryl C. Zeldin, NIH, USA)^48^, CD31 (1:100, DIA-310, Dianova) and α​-smooth muscle actin–cy3 (1:500, C6198 Sigma). For secondary detection, Alexa Fluor-coupled secondary antibodies (1:200, Invitrogene) were used. Cell nuclei were visualized with DAPI (0.2 μ​g ml^−1^, D9542 Sigma). Rabbit IgG (1:250, sc-2027, Santa Cruz, Germany) served as negative control for sEH. Samples were visualized using a confocal microscope (LSM 780 scanning confocal microscope; Zeiss, Jena, Germany).

### Western blot

OA tissues (n = 5–6 mice per pool per replicate) were homogenized on ice in RIPA lysis buffer (50 mmol/L Tris–HCl pH 7.5, 150 mol/L NaCl, 10 mol/L NaPPi, 20 mol/L NaF, 1% sodium deoxycholate, 1% Triton X-100 and 0.1% SDS) and detergent-soluble proteins were resuspended in SDS–PAGE sample buffer. Samples were separated by SDS–PAGE and subjected to western blotting, as described^35^. Membranes were blocked in 3% BSA in TBS, incubated with primary and horseradish peroxidase-conjugated secondary antibodies in blocking solution, and detection was performed with a Lumi-Light plus western blotting substrate (Roche). Antibodies against 2C44 (1:1000), sEH (1:1000) and β-actin (1:5000, A5316 Sigma) were used. Secondary antibodies were used at a dilution of 1:10,000.

### Determination of EETs by liquid chromatography-tandem mass spectrometry (LC–MS/MS)

The determination of EETs was carried out as described elsewhere^74^. Briefly, the following standard compounds and their corresponding deuterated derivatives were obtained from Cayman Chemicals (Ann Arbor, MI, USA): 5,6-EET, 8,9-EET, 11,12-EET, 14,15-EET, 5,6-EET-d11, 8,9-EET-d11, 11,12-EET-d11 and 14,15-EET-d11. Prior to the lipid extraction, tissue samples were homogenized using a Mixer-mill MM400 and grinder zirconium balls (Ø 0.5 mm) from Retsch (Haan, Germany). Stock solutions containing 25,000 ng/ mL of all analytes were prepared in methanol. These solutions were further diluted also with methanol to obtain working solutions. The calibration curve ranged from 0.5 to 3000 ng/ mL for EET and from 0.25 to 1500 ng/mL for DHET. Sample extraction was performed using liquid–liquid extraction. Therefore, tissues were extracted twice with 600 µl of ethyl acetate. The combined organic phases were removed at a temperature of 45 °C under a gentle stream of nitrogen. The residues were reconstituted in 50 µL of methanol: water: BHT (50: 50: 10^−4^, v/v/v) prior to injection into the LC–MS/MS system.

The chromatographic separation and lipid quantification were performed as described previously^74^. The LC–MS/MS system consisted of a QTrap 5500 from Sciex (Darmstadt, Germany) equipped with a Turbo-V source operating in negative electrospray ionization mode, an Agilent 1260 binary HPLC pump and degasser (Waldbronn, Germany), and an HTC Pal autosampler (CTC Analytics, Zwingen, Switzerland) fitted with a 25 µL LEAP syringe (Axel Semrau, Sprockhövel, Germany). High-purity nitrogen for the mass spectrometer was produced by a NGM 22-LC–MS nitrogen generator (cmc Instruments, Eschborn, Germany). For the chromatographic separation, a Gemini NX C18 column and precolumn were used (150 × 2 mm ID, 5 µm particle size, and 110 Å pore size; Phenomenex, Aschaffenburg, Germany). A linear gradient was used at a flow rate of 0.5 mL/ min with a total run time of 17.5 min. Mobile phase A was water: ammonia (100: 0.05, v/v), and mobile phase B was acetonitrile: ammonia (100: 0.05, v/v). The gradient started at 85% A and changed to 10% A within 12 min. This setup was held for 1 min. Within 0.5 min, the gradient shifted back to 85% A and was held for 4 min to re-equilibrate the column. The injection volume was 20 µL.

All data were acquired using Analyst software v1.6.2 and quantitation was performed by MultiQuant software v3.0 (both Sciex, Darmstadt, Germany) using the internal standard method (isotope-dilution mass spectrometry). Calibration curves were calculated by linear regression with 1/x weighting.

### Dissection and preparation of microvessels

The preparation of the arterial segments from mice and vascular reactivity studies were carried out as described elsewhere^3,71^. Briefly, the ophthalmic artery was meticulously isolated and dissected into rings of 3–5 mm in length. The arterial rings were placed in an organ bath with ice-cold Krebs–Henseleit buffer, cannulated, pressurized to 50 mm Hg and equilibrated prior to the commencement of the experiments. The organ bath was continuously circulated with Krebs buffer aerated with a gaseous mixture of 95% O_2_ and 5% CO_2,_ while the temperature was maintained at 37 °C and pH 7.4. Vessel viability was assessed as a minimum of 50% vasoconstriction from resting diameter in response to 100 mmol/L KCl. For assessment of vascular function, the rings were pre‐contracted with phenylephrine to reach 70–50% of the initial vessel diameter, before vasodilation was induced with ACh. All reported drug concentrations represent the final molar concentrations in the organ bath. Solutions were prepared fresh daily and discarded after each experiment. Unless otherwise stated, all experiments were carried out in the presence of L-NAME (100 µmol/L), indomethacin (10 µmol/L) and baicalein (10 µmol/L) to prevent the influence of endogenous nitric oxide, prostacyclin and lipooxygenase, respectively. Video sequences of the vessel reactivity were captured to a personal computer using a video camera mounted on an inverted microscope for off-line analyses.

### Experimental protocols for vasoreactivity study

The overview of the inhibitors employed in the present study to investigate the vasoreactivity of the arachidonic acid metabolites in the murine ophthalmic artery is depicted as a scheme (Supplementary Fig. S3 online).

#### Protocol 1

To investigate the functional role of specific enzymes in the arachidonic acid pathway in mediating the vasoreactivity of the ophthalmic artery, responses of phenylephrine-pre-constricted vessels to cumulative administration of ACh (1 nmol/L–100 µmol/L) were tested before and after 30 min of incubation with the following inhibitors: Quinacrine [Phospholipase A_2_ (PLA_2_) inhibitor; 10 µmol/L], Sulfaphenazole (specific CYP2C epoxygenase inhibitor; 10 µmol/L), MS-PPOH (CYP epoxygenase inhibitor; 10 µmol/L), HET0016 (selective ω-hydroxylase inhibitor; 1 µmol/L), AUDA and TPPU (sEH inhibitors; 100 nmol/L) and 14,15-EEZE (EET antagonist; 10 µmol/L). Our previous study has demonstrated that 17-ODYA exerted significant blunting of vasodilation in the murine ophthalmic artery^3^. However, 17-ODYA is a well-known non-specific suicide substrate inhibitor, which inhibits both ω-hydroxylation and epoxygenation of arachidonic acid with equal potency^10,75^. Hence, to determine the precise contribution of the metabolites generated via these two pathways, selective blockers for epoxygenases and ω-hydroxylases were employed. To further characterize the role of Cyp epoxygenase-derived metabolites of arachidonic acid on the vasoreactivity of the ophthalmic artery, the effect of inhibiting the main metabolizing enzyme, soluble epoxide hydrolase (sEH), was investigated. The inhibition of sEH prolongs the half-life of endogenous EETs and thereby, increases the bioavailability of latter in the artery. However, the efficacy of certain inhibitors is highly dependent on the relative sensitivity of the vasculature to the agent used. Since this is the first study to assess the utility of sEH inhibitors in the ophthalmic artery, the use of one particular inhibitor alone may not be able provide a clear understanding of the role of sEH. Therefore, two structurally dissimilar but highly potent sEH inhibitors comprising AUDA and TPPU^6,45–47^ were employed to further validate the role of sEH in modulating vascular responses and to determine whether the attenuated metabolism of EETs to their corresponding dihydroxy derivatives (DHETs) as a function of sEH inhibition would affect the vasodilatory profile of this artery. Subsequently, to confirm that the vasomotor tone regulation of the ophthalmic artery is mediated by EETs, vasodilation to ACh was assessed in the absence and presence of 14,15-EEZE, which is a direct, non-selective antagonist of EETs^7,24,45^.

#### Protocol 2

Subsequently, the second protocol was designed to identify the effect of each regioisomer of EETs in the ophthalmic artery. To achieve this objective, exogenous application (0.01 nmol/L–1 µmol/L) of synthetic EETs (5,6-, 8,9-, 11,12- and 14,15-EETs) was separately administered to pre-constricted vessels with and without incubation with AUDA (100 nmol/L). At the end of each experiment with each EET regioisomer, the organ chambers were rinsed with at least six exchanges of fresh Krebs buffer to remove any residual EET, which had not been taken up by the arterial tissues.

### Statistics

Statistical analysis was carried out as described in our previous studies^3,71^. Data are expressed as mean ± SEM, with n representing the number of animals per group. Changes in vascular responses to various reagents tested are presented as percentage of diameter change from the initial precontraction levels or the percent vasodilator responses as compared to maximal vasodilator response induced by ACh. Statistical comparisons of concentration- response curves were made using the two-way ANOVA for repeated measures followed by Bonferroni post-hoc test. Unpaired two-tailed t-test was used for single-dose responses. The level of significance α was set at 0.05. Graph Pad Prism 6 software (GraphPad Inc., San Diego, USA) was used for statistical analyses.

## Supplementary Information

Supplementary Information.
