# Supplementary material for: Cyp2c44 epoxygenase-derived epoxyeicosatrienoic acids in vascular smooth muscle cells elicit vasoconstriction of the murine ophthalmic artery
Source: Sci Rep. 2021 Sep 21;11:18764. doi: 10.1038/s41598-021-98236-w (PMC8455677; doi:10.1038/s41598-021-98236-w)
Supplement: Supplementary file 1 — Supplementary Information. [file 41598_2021_98236_MOESM1_ESM.pdf]

SUPPLEMENTARY FILE

Cyp2c44 epoxygenase-derived epoxyeicosatrienoic acids in vascular smooth muscle cells elicit vasoconstriction of the murine ophthalmic artery

Jiong Hu[b,c], Marco Sisignano[d,e], Roman Brecht[a], Natarajan Perumal[a], Carlo Angioni[d], Iris-Sofia Bibli[b,c], Beate Fisslthaler[b,c], Hartmut Kleinert[f], Norbert Pfeiffer[a], Ingrid Fleming[b,c], Caroline Manicam[a*]

[a]Department of Ophthalmology, University Medical Centre of the Johannes Gutenberg University Mainz, Mainz, Germany.
[b]Institute for Vascular Signalling, Centre for Molecular Medicine, Goethe University, Frankfurt am Main, Germany.
[c]German Centre of Cardiovascular Research (DZHK), Partner site RheinMain, Frankfurt am Main, Germany.
[d]Pharmazentrum Frankfurt/ZAFES, Institute of Clinical Pharmacology, University Hospital, Goethe-University, Frankfurt am Main, Germany.
[e]Fraunhofer Institute for Translational Medicine and Pharmacology ITMP, Frankfurt am Main, Germany.
[f]Department of Pharmacology, University Medical Centre of the Johannes Gutenberg University Mainz, Mainz, Germany.

***Corresponding author:**
Caroline Manicam, Ph.D,
Department of Ophthalmology,
University Medical Centre of the Johannes Gutenberg University Mainz,
Langenbeckstr. 1,
55131 Mainz, Germany.
E-mail: caroline.manicam@unimedizin-mainz.de
Tel: +49 6131 17-8276

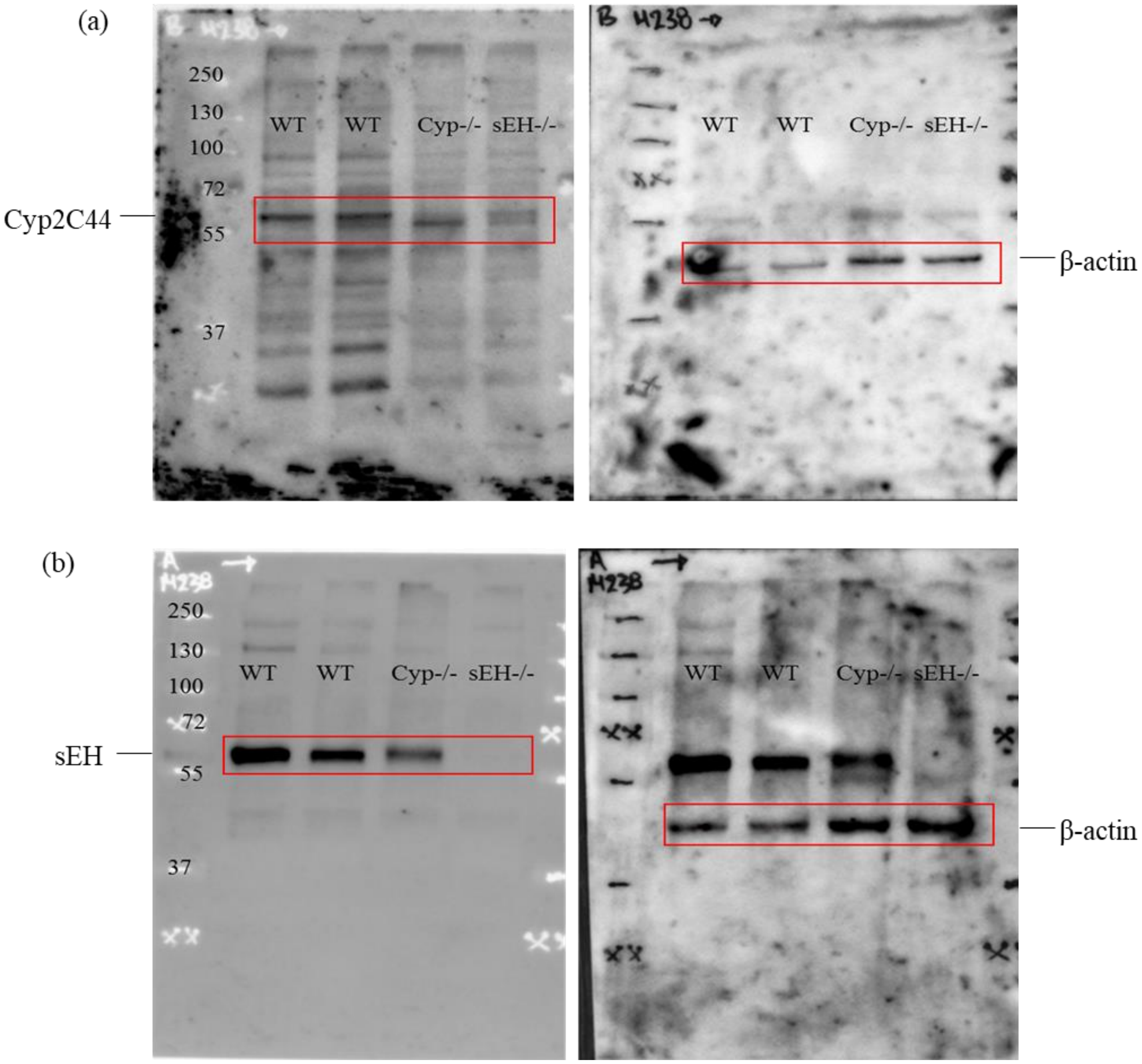

Supplementary Fig. S1. Original Western blot gels.
Full gel pictures showing the bands that correspond to the expression of (a) Cyp2c44 and (b) sEH corresponding β-actin loading control. Samples were pooled from n= 5 mice/ WT and n= 6 mice/ Cyp2c44-/- and sEH-/- for each lane, per Western blot analysis.

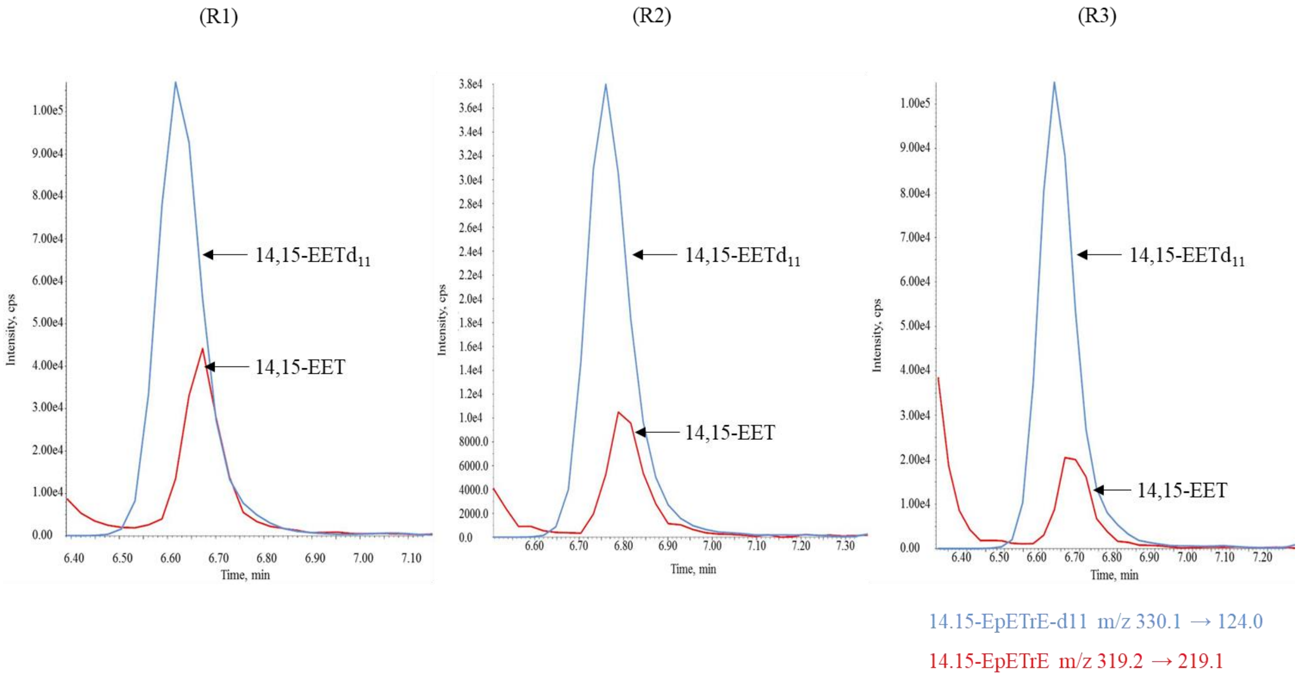

Supplementary Fig. S2. Chromatograms of 14,15-EET in the murine ophthalmic artery. Representative LC-MS/MS chromatogram of 14, 15-EET (in red) compared to deuterated internal standard (in blue). R1- R3: replicate 1-3; n = 5/ replicate. cps: count per second.

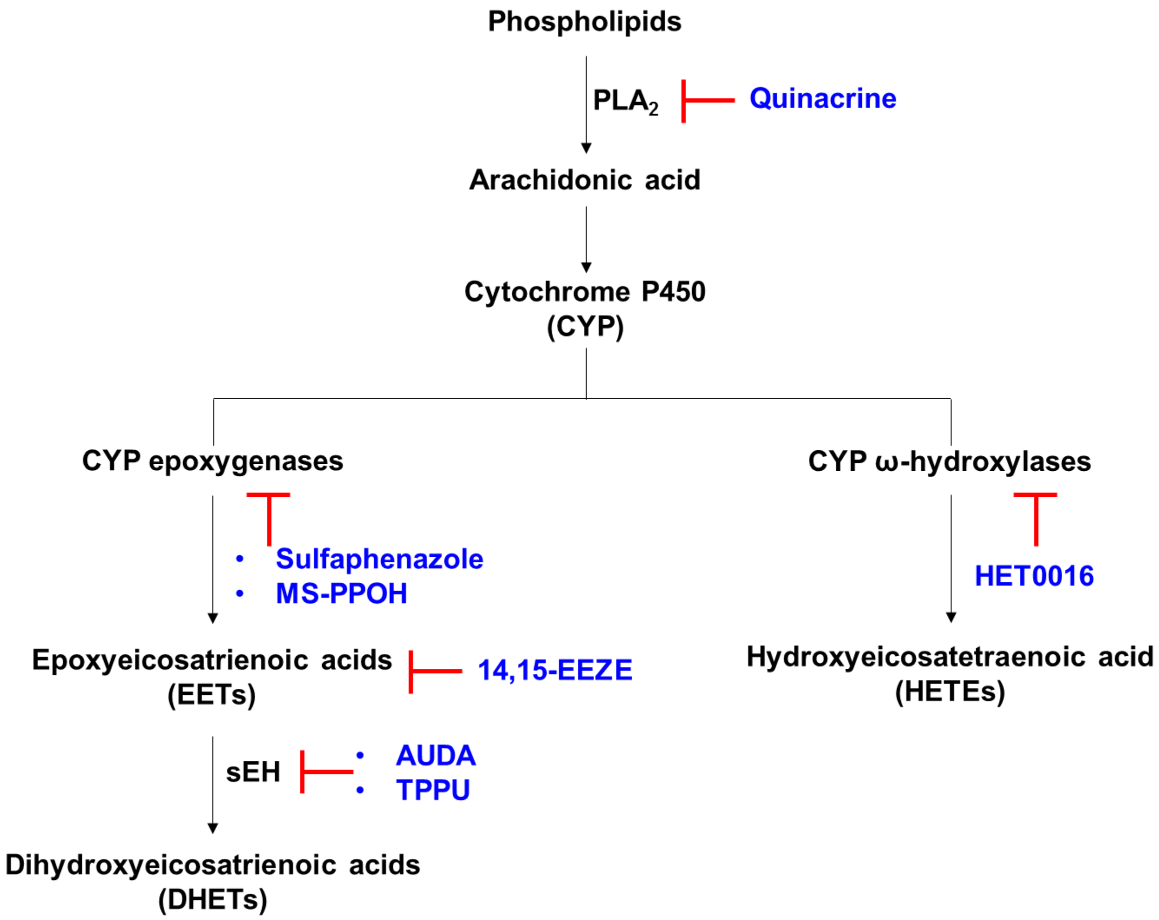

Supplementary Fig. S3. Overview of various pharmacological tools employed in this study to investigate the contribution of the arachidonic acid signalling mediators in the murine ophthalmic artery.

Phospholipase A2 (PLA2) releases arachidonic acid from the cell plasma membrane, which is then metabolized by CYP enzymes to produce four regioisomers of epoxyeicosatrienoic acids (EETs) via the CYP epoxygenase pathway or hydroxyeicosatetraenoic acid (HETEs) via the CYP ω-hydroxylase pathway. Epoxyeicosatrienoic acids are catalysed by soluble epoxide hydrolase (sEH) to corresponding vicinol diols, dihydroxyeicosatrienoic acids (DHETs). Two structurally distinct inhibitors of CYP and sEH comprising MS-PPOH and sulfaphenazole to inhibit the former, and AUDA and TPPU to inhibit the latter, were used to examine their effects on ACh-induced vasorelaxation.
